# Elevated HDL Levels Linked to Poorer Cognitive Ability in Females With Parkinson’s Disease

**DOI:** 10.3389/fnagi.2021.656623

**Published:** 2021-06-11

**Authors:** Megan C. Bakeberg, Anastazja M. Gorecki, Jade E. Kenna, Alexa Jefferson, Michelle Byrnes, Soumya Ghosh, Malcolm K. Horne, Sarah McGregor, Rick Stell, Sue Walters, Frank L. Mastaglia, Ryan S. Anderton

**Affiliations:** ^1^Perron Institute for Neurological and Translational Science, Nedlands, WA, Australia; ^2^Centre for Neuromuscular and Neurological Disorders, The University of Western Australia, Perth, WA, Australia; ^3^School of Biological Sciences, The University of Western Australia, Perth, WA, Australia; ^4^The Florey Institute of Neuroscience and Mental Health, University of Melbourne, Parkville, VIC, Australia; ^5^Centre for Clinical Neurosciences and Neurological Research, St Vincent’s Hospital Melbourne, Fitzroy, VIC, Australia; ^6^School of Health Sciences, Institute for Health Research, The University of Notre Dame Australia, Fremantle, WA, Australia

**Keywords:** Parkinson’s disease, cognitive decline, cognitive impairment, domain-specific, cholesterol, HDL-cholesterol, sex-specific

## Abstract

**Introduction:**

Cholesterol levels have been associated with age-related cognitive decline, however, such an association has not been comprehensively explored in people with Parkinson’s disease (PD). To address this uncertainty, the current cross-sectional study examined the cholesterol profile and cognitive performance in a cohort of PD patients.

**Methods:**

Cognitive function was evaluated using two validated assessments (ACE-R and SCOPA-COG) in 182 people with PD from the Australian Parkinson’s Disease Registry. Total cholesterol (TC), high-density lipoprotein (HDL), low-density lipoprotein (LDL), and Triglyceride (TRG) levels were examined within this cohort. The influence of individual lipid subfractions on domain-specific cognitive performance was investigated using covariate-adjusted generalised linear models.

**Results:**

Females with PD exhibited significantly higher lipid subfraction levels (TC, HDL, and LDL) when compared to male counterparts. While accounting for covariates, HDL levels were strongly associated with poorer performance across multiple cognitive domains in females but not males. Conversely, TC and LDL levels were not associated with cognitive status in people with PD.

**Conclusion:**

Higher serum HDL associates with poorer cognitive function in females with PD and presents a sex-specific biomarker for cognitive impairment in PD.

## Introduction

Parkinson’s disease (PD) is the world’s second most common neurological disease and is associated with a variety of motor and non-motor symptoms. Non-motor symptoms, including cognitive impairment and dementia, are commonly reported as being equally as debilitating as cardinal motor symptoms (Triantafyllou et al., 2008; Tarolli et al., 2019). Longitudinal studies indicate that nearly 50% of people with PD develop some degree of mild cognitive impairment (MCI) after 10 years of the disease. This proportion rises to over 80% after 20 years of disease course, which is particularly disturbing as PD-MCI itself is associated with impaired quality of life, increased caregiver burden, and increased risk of progression to dementia (PDD) (Hely et al., 2008; Aarsland and Kurz, 2010; Cosgrove et al., 2015). As such, developing objective biological measures associated with the progression of disease-associated cognitive decline will greatly assist in clinical practice.

As a well-established risk factor for cerebrovascular and cardiovascular disease (CVD) (Goldstein and Brown, 2015; Leritz et al., 2016; Mansoor et al., 2016a), cholesterol profiles and metabolism are being increasingly implicated in age-related cognitive impairment, vascular dementia, and Alzheimer’s disease. Interestingly, within the context of PD, a number of studies have indicated that higher serum cholesterol levels are associated with reduced risk of PD, and that certain lipid subfractions are lower in individuals with PD when compared to healthy controls (Huang et al., 2011; Rozani et al., 2018; Kummer et al., 2019; Choe et al., 2021). Serum cholesterol is comprised of a number of lipid subfractions including low-density lipoproteins (LDLs) and high-density lipoproteins (HDLs). When in excess, unused LDL subfractions are commonly deposited in the arteries (Goldstein and Brown, 2015; Mansoor et al., 2016a,b) and for this reason have been associated with poor cardiovascular outcomes. However, in more recent larger studies, lower LDL levels have been associated with age-associated dementia, indicating a possible protective effect of this form of cholesterol (Zhou et al., 2018).

In contrast, HDL is thought to have anti-oxidative, anti-inflammatory and cardioprotective capabilities (Hottman et al., 2014). For instance, some studies have tied elevated HDL levels with superior cognitive function (Henderson et al., 2003; Reitz et al., 2010; Crichton et al., 2014; Svensson et al., 2019) and low HDL levels being associated with decreased hippocampal volume (Wolf et al., 2004; Ledreux et al., 2016). A recent cross-sectional study investigated the relationship between serum lipid profile and cognitive function in healthy ageing women, with elevated HDL level being identified as a marker for improved cognition, better verbal memory and superior learning ability (Bates et al., 2017). While associated with disease risk of a number of neurodegenerative diseases (Martín et al., 2014), there is a paucity of studies of this nature within the context of cognitive decline in PD. While one recent study examined the association between lipid levels and PD-related symptoms, no significant associations were noted (Choe et al., 2021). However, an increasing body of literature indicates notable sex-differences among various aspects of the clinical presentation of PD (Augustine et al., 2015; Liu et al., 2015; Bakeberg et al., 2020a), and cognitive impairment specifically (Bakeberg et al., 2019, 2021). Thus, it is of utmost importance to examine these associations in light of such sex-differences.

As an individual’s lipid profile is known to be a relatively modifiable target for intervention and prevention through non-invasive means, coupled with growing evidence implicating cholesterol levels in cognition, exploring the association between lipid profile and cognitive impairment in PD is of major clinical significance and therapeutic potential. Although an individual’s sex is identified to significantly change the trajectory of cognitive decline (Song et al., 2014; Nicoletti et al., 2017; Cholerton et al., 2018; Reekes et al., 2020), it is often disregarded (Leritz et al., 2016; de Oliveira et al., 2017), and rarely controlled for Reitz et al. (2010); Crichton et al. (2014), Sterling et al. (2016); Svensson et al. (2019). Here, we focussed on cholesterol markers (TC, LDL, and HDL levels) that have previously been examined in the context of cognitive impairment, and whether or not there was a sex-specific effect of these lipid fractions on cognitive ability in people with PD.

## Materials and Methods

### Participants

One hundred and eighty-two community-based individuals with PD (114 males and 68 females) were sequentially recruited, as previously described (Evans et al., 2017; Riley et al., 2018; Bakeberg et al., 2019). In brief, participants were recruited from Movement Disorders Clinics at the Perron Institute for Neurological and Translational Science (Perth, WA, Australia) and St. Vincent’s Hospital (Melbourne, VIC, Australia), between 2012 and 2015. All individuals with PD were examined by a movement disorder neurologist prior to inclusion in the study for verification of the diagnosis in accordance with the United Kingdom Brain Bank criteria for idiopathic PD (Hughes et al., 1992). This study was approved by a Human Research and Ethics Committee (Approval number 2006/073), and written informed consent was obtained from all participants, in accordance with the National Health and Medical Research Council guidelines.

### Clinical Assessments of Participants

Clinical evaluations included collection of patient demographic variables and medication dosage, assessments of motor and cognitive function, and other disease-related features (Table 1). All PD medications were converted to a total levodopa equivalent daily dose (LEDD), based on a previously reported conversion equation (Parkin et al., 2002; Tomlinson et al., 2010). Motor symptoms were evaluated in the “ON” state using the Movement Disorder Society-Unified Parkinson’s Disease Rating Scale (MDS-UPDRS) Part III (Goetz et al., 2007). In addition, each participant was evaluated by a clinical psychologist and completed a panel of neuropsychological assessments, as previously described (Evans et al., 2017; Bakeberg et al., 2019). Briefly, global cognitive function was assessed using the Scales of Outcomes in Parkinson’s disease – Cognition (SCOPA-Cog), and global and domain-specific cognition was also assessed using the revised “Addenbrooke’s Cognitive Examination” (ACE-R 2004). The SCOPA-Cog is a reliable and validated tool to assess cognitive function, specifically within PD (Marinus et al., 2003), whereby scores can range between 0 and 43, with higher scores representing superior cognitive performance. Studies have determined that scores of 30 or less may be considered accurately representative of MCI (Marras et al., 2013). Whereas, the ACE-R provides an evaluation of global cognitive function, as well as domain-specific assessment of attention and orientation, memory, verbal fluency, language and visuospatial-perceptual ability (Mioshi et al., 2006; Bakeberg et al., 2020b). Cut-off ACE-R scores of ≤88 out of 100 have been used previously as an indicator for probable cases of MCI (Marras et al., 2013).

**TABLE 1:** Clinical characteristics of the PD cohort used in this study, when combined and when compared by sex.

		**Mean (SD) or *n* (%)**			
**Clinical characteristics**		**Combined (*n* = 182)**	**Males (*n* = 114)**	**Females (*n* = 68)**	**Significance ^a^ (*p-*value; Cohen’s *d*)**
Age (years)		64.5 (9.5)	64.9 (10.4)	63.9 (7.8)	*p* = *0.236; d* = 0.11
Age at onset (years)		55.4 (10.5)	55.4 (11.4)	55.3 (8.8)	*p* = *0.997; d* = 0.01
Disease duration (years)		9.2 (6.0)	9.5 (6.4)	8.6 (5.6)	*p* = *0.473; d* = 0.15
LEDD (mg/day)		942.1 (622.6)	932.9 (618.4)	957.3 (633.7)	*p* = *0.883; d* = 0.04
DBS	Yes	26 (14.3%)	19 (16.7%)	7 (10.3%)	***p* < *0.001***
	No	156 (85.7%)	95 (83.3%)	61 (89.7%)	
DA	Yes	87 (47.8%)	52 (45.6%)	32 (47.1%)	*p* = *0.603*
	No	94 (51.6%)	63 (54.4%)	35 (51.5%)	
MDS-UPDRS	III	20.6 (14.9)	22.5 (14.7)	17.4 (14.7)	***p* = *0.005****; d* = 0.35
ACE-R	Total	85.3 (13.4)	84.2 (14.7)	87.0 (11.0)	*p* = *0.274; d* = 0.22
SCOPA-Cog	Total	26.5 (8.1)	25.8 (8.4)	27.3 (7.6)	*p* = *0.324; d* = 0.19

*^*a*^Independent Samples test, Mann–Whitney *U* test or Spearman’s Chi Square test conducted for between group analyses, based on sex.*

*LEDD, Levodopa Equivalent Daily Dose; DBS, deep brain stimulation; DA, dopamine agonists; MDS-UPDRS III, Movement Disorder Society-Unified Parkinson’s Disease Rating Scale III; ACE-R, Addenbrooke’s Cognitive Examination – Revised; SCOPA-Cog, Scales for Outcomes in Parkinson’s Disease Cognition.*

*Bolding to highlight statistical significance. These italic values are stylistic.*

### Serum Analysis

Fasted participant blood samples were collected prior to clinical and psychological assessments. For blood collection, 10 ml of whole blood was taken from a median cubital vein, and stored in a standard BD EDTA vacutainer^®^ (Becton Dickinson and Company, Franklin Lakes, NJ, United States). Serum TC, LDL, HDL, and triglyceride (TRG) levels were assessed via routine lipid biochemistry, as conducted at State Pathology centres. Normal/recommended serum subfraction levels are as follows: TC, 2.9–5.5 mmol/L; LDL, 1.7–3.5 mmol/L; HDL, 1.0–2.9 mmol/L; and TRG, 0.2–2.0 mmol/L (Tonkin et al., 2005; Bates et al., 2017).

### Statistical Methods

Data was analysed using IBM-SPSS (v. 26, IBM Corporation). Variables were described using mean ± standard deviation (SD), or frequency and percent (%), as appropriate. A significant nominal *p*-value of <0.05 was employed for all statistical tests. Continuous variables distributions were assessed using the Shapiro–Wilk test of normality. Where appropriate, univariate regression analysis or Mann–Whitney *U* analysis was performed to identify differences between groups. Cohen’s *d* ES were calculated for the mean differences, with an ES of 0.20 considered small, 0.50 medium and 0.80 large.

To investigate associations between cholesterol and cognitive scores, male and female participants were analysed separately. Generalised linear models (GLMs) were used as univariate models to investigate association between cognitive outcome measures and cholesterol serum markers. Multivariable adjusted GLMs were used for analysing relationships between lipids and cognitive function, while taking covariates into account. Variables considered as covariates included those identified in grouped analyses (being DBS and MDS-UPDRS III), as well as other variables known to influence cognitive performance, such as age at time of assessment, age of onset, disease duration, and parkinsonian medications. All variables were included in GLMs to determine whether they were significantly associated with cognitive outcome measures, prior to inclusion as a covariate in multivariable GLMs. Residual plots were examined for all models and no violations were noted.

## Results

### Cohort Information

Clinical characteristics of the PD cohort are summarised in Table 1. This cohort was predominantly male (62.6%), with mean age at assessment of 64.5 years, mean disease duration of 9.2 years and mean age at PD onset of 55.4 years, and these did not vary significantly when separated by sex. Males had a significantly higher mean MDS-UPDRS part III score (22.6 ± 15 vs. 17.4.9 ± 15; *p* = 0.005, males vs. females) and were also more likely to have received DBS treatment (16.7% vs. 10.3%; *p* = 0.001) (Table 1). There were no significant sex-specific differences in PD medication treatment, specifically no differences in LEDD or DA dosage. Importantly, there were no mean differences in cognitive performance between male and female cohorts, as determined by ACE-R or SCOPA-Cog assessments (Table 1). Subsequent analyses of relationships between lipids and cognitive function therefore included MDS-UPDRS III scores and DBS status as covariates, as well as other variables which influence cognitive performance (age at assessment, age of PD onset, and disease duration).

### Sex-Based Differences Within Lipid Profile of PD Cohort

To explore the association of lipids with cognitive performance in light of recognised sex differences in lipid profiles, a total of 114 males and 68 females with PD were included. When examining mean lipid profiles of the whole cohort, TC was 5.1 mmol/L (±1.0), LDL was 3.1 (±0.9), HDL levels were 1.5 (±0.4), and TRG was 1.1 mmol/L (±0.5); all of which fall within normal reported ranges of serum lipid profile levels (TC, 2.9–5.5 mmol/L; LDL, 1.7–3.5 mmol/L; HDL, 1.0–2.9 mmol/L; and TRG, 0.2–2.0 mmol/L) (Tonkin et al., 2005; Bates et al., 2017).

**FIGURE 1:**
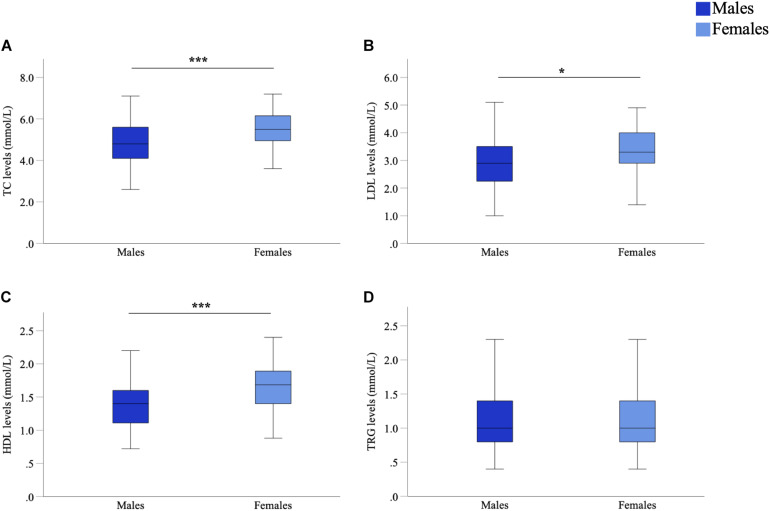
Comparison of serum marker levels between males and females in a Parkinson’s disease (PD) cohort. Mean TC **(A)**, LDL **(B)**, and HDL **(C)** levels were significantly higher in females than males, mean TRG **(D)** levels were not significantly different. Data are presented as mean ± SEM. *p < 0.05; ***p < 0.001. TC, total cholesterol; LDL, low-density lipoprotein; HDL, high-density lipoprotein; TRG, triglyceride.

As sex-specific differences in lipid profiles are often reported in healthy populations, we compared sex differences in lipid profiles of this cohort of people with PD. We observed significant differences between male and female participants in TC (*p* < 0.001, *d* = 0.62), LDL (*p* = 0.012, *d* = 0.35) and HDL (*p* < 0.001, *d* = 0.83) levels (Figure 1). Such a difference could not be said for TRG levels (*p* = 0.455, *d* = 0.06) (Figure 1D). Specifically, males had lower mean lipid levels than females for TC (4.92 vs. 5.53 mmol/L, male vs. female), LDL (3.01 vs. 3.33 mmol/L) and HDL levels (1.40 vs. 1.71 mmol/L). As such, to accurately explore the effects of lipids on cognitive performance in people with PD, separated analyses based upon participant sex was considered imperative and were employed for further statistical analyses concerning TC, LDL and HDL levels. Whereas, analysis of TRG levels was conducted with males and females being combined.

### Cognitive Performance and TC Levels

Regardless of sex, levels of TC were not significantly associated with measures of cognition (total scores or subdomains) in naïve GLMs in most instances, although there was a significant association within the domain of attention and orientation in female participants (*p* = 0.030; Table 2). When considering covariates, TC was significantly associated with the ACE-R subdomains of attention and orientation (*p* = 0.017) and visuospatial domain (*p* = 0.011) within females with PD (Table 2). Conversely, TC levels were not significantly associated with cognitive status in males, in either unadjusted or covariate adjusted comparisons. Whereas, in females, certain significant associations were evident between TC levels and cognitive status in adjusted comparisons.

**TABLE 2:** Cognitive performance in participants based on TC levels, when separated by sex.

**Variable**	**Unadjusted**		**Adjusted**	
**Males (*n* = 114)**	**β-Co (SEM)**	***p*^*a*^ value**	**β-Co (SEM)**	***p*^b^ value**
Total ACE-R	1.678	0.227	0.693	0.611
Attention and orientation	0.121	0.506	–0.039	0.822
Memory	0.521	0.348	0.184	0.734
Fluency	0.554	0.154	0.385	0.310
Language	0.212	0.338	0.089	0.689
Visuospatial	0.173	0.575	0.043	0.891
Total SCOPA-Cog	0.837	0.273	0.312	0.687

**Females (*n* = 68)**	**β-Co (SEM)**	***p*^*a*^ value**	**β-Co (SEM)**	***p*^b^ value**

Total ACE-R	–1.253	0.374	–2.086	0.135
Attention and orientation	–0.543	**0.030**	–0.611	**0.017**
Memory	–0.050	0.938	–0.567	0.382
Fluency	0.092	0.844	–0.096	0.844
Language	–0.282	0.231	–0.165	0.448
Visuospatial	–0.473	0.088	–0.650	**0.011**
Total SCOPA-Cog	–0.531	0.586	–0.616	0.500

*^*a*^*p-*value taken from GLM without adjustment for covariates.*

*^*b*^*p-*value taken from GLM adjusted for DBS, MDS-UPDRS III, age at time of assessment, age of onset and disease duration.*

*ACE-R, Addenbrooke’s Cognitive Examination – Revised; SCOPA-Cog, Scales for Outcomes in Parkinson’s Disease Cognition.*

*Bolding to highlight statistical significance.*

### Cognitive Performance and LDL Levels

Levels of LDL were not significantly associated with any measure of cognition in naïve GLMs, regardless of sex (Table 3). However, LDL levels of females with PD were significantly associated with the visuospatial domain of the ACE-R assessment, in a model adjusting for covariates (*p* = 0.013). On the other hand, LDL levels were not significantly associated with cognitive status of males, in either unadjusted or adjusted comparisons.

**TABLE 3:** Cognitive performance in participants based on LDL levels, when separated by sex.

**Variable**	**Unadjusted**		**Adjusted**	
**Males (*n* = 114)**	**β-Co (SEM)**	***p*^*a*^ value**	**β-Co (SEM)**	***p*^b^ value**
Total ACE-R	2.150	0.164	1.187	0.433
Attention and orientation	0.204	0.311	0.065	0.736
Memory	0.645	0.297	0.275	0.648
Fluency	0.557	0.199	0.396	0.349
Language	0.324	0.188	0.214	0.389
Visuospatial	0.283	0.410	0.169	0.631
Total SCOPA-Cog	1.068	0.207	0.515	0.550

**Females (*n* = 68)**	**β-Co (SEM)**	***p*^*a*^ value**	**β-Co (SEM)**	***p*^b^ value**

Total ACE-R	0.204	0.891	–0.648	0.667
Attention and orientation	–0.276	0.308	–0.311	0.271
Memory	0.453	0.500	–0.131	0.850
Fluency	0.423	0.387	0.248	0.633
Language	0.066	0.792	0.224	0.330
Visuospatial	–0.461	0.115	–0.676	**0.013**
Total SCOPA-Cog	0.569	0.580	0.418	0.668

*^*a*^*p-*value taken from GLM without adjustment for covariates.*

*^*b*^*p-*value taken from GLM adjusted for DBS, MDS-UPDRS III, age at time of assessment, age of onset and disease duration.*

*ACE-R, Addenbrooke’s Cognitive Examination – Revised; SCOPA-Cog, Scales for Outcomes in Parkinson’s Disease Cognition.*

*Bolding to highlight statistical significance.*

### Cognitive Performance of Females, but Not Males, Is Significantly Affected by HDL Levels

In males, univariate analyses revealed HDL was not associated with cognition scores. In models adjusted for covariates, there remained no association between HDL and cognitive scores (Table 4). In contrast, in females, univariate analysis revealed HDL levels were significantly associated with ACE-R subdomains, attention and orientation, memory, fluency, language and total SCOPA-Cog, but not with visuospatial cognition. In models adjusting for covariates, HDL levels in females were significantly associated with total ACE-R score (*p* = 0.001), ACE-R subdomain scores of attention and orientation (*p* = 0.001), memory (*p* = 0.026), fluency (*p* = 0.023) and language (*p* < 0.001); and total SCOPA-Cog score (*p* = 0.004). However, the visuospatial domain did not exhibit a significant association with HDL levels in females, regardless of covariate adjustment (*p* = 0.351, Table 4).

**TABLE 4:** Cognitive performance in participants based on HDL levels, when separated by sex.

**Variable**	**Unadjusted**		**Adjusted**	
**Males (*n* = 114)**	**β-Co (SEM)**	***p*^*a*^ value**	**β-Co (SEM)**	***p*^b^ value**
Total ACE-R	0.808	0.849	–1.169	0.776
Attention and orientation	–0.222	0.687	–0.584	0.261
Memory	0.664	0.695	–0.076	0.963
Fluency	1.468	0.215	0.857	0.454
Language	–0.416	0.538	–0.428	0.524
Visuospatial	–0.229	0.808	–0.470	0.622
Total SCOPA-Cog	1.670	0.489	0.848	0.712

**Females (*n* = 68)**	**β-Co (SEM)**	***p*^a^ value**	**β-Co (SEM)**	***p*^b^ value**

Total ACE-R	–9.998	**0.001**	–9.571	**0.001**
Attention and orientation	–1.781	**0.002**	–1.900	**0.001**
Memory	–3.187	**0.029**	–3.165	0.026
Fluency	–2.667	**0.011**	–2.401	**0.023**
Language	–1.779	**0.001**	–2.053	**<0.001**
Visuospatial	–0.594	0.371	–0.558	0.351
Total SCOPA-Cog	–5.879	**0.007**	–5.545	**0.004**

*^*a*^*p*-value taken from GLM without adjustment for covariates.*

*^*b*^*p-*value taken from GLM adjusted for DBS, MDS-UPDRS III, age at time of assessment, age of onset and disease duration.*

*ACE-R, Addenbrooke’s Cognitive Examination – Revised; SCOPA-Cog, Scales for Outcomes in Parkinson’s Disease Cognition.*

*Bolding to highlight statistical significance.*

### Cognitive Performance and TRG Levels

Levels of TRG were not significantly associated with any measure of cognition in both unadjusted and adjusted GLMs (Table 5).

**TABLE 5:** Cognitive performance in participants based on TRG levels, not separated by sex.

**Variable**	**Unadjusted**		**Adjusted**	
**Males and Females (*n* = 182)**	**β-Co (SEM)**	***p*^a^ value**	**β-Co (SEM)**	***p*^b^ value**
Total ACE-R	0.466	0.825	0.287	0.888
Attention and orientation	0.047	0.876	0.029	0.922
Memory	–0.020	0.982	–0.069	0.935
Fluency	0.251	0.685	0.272	0.655
Language	–0.140	0.681	–0.161	0.631
Visuospatial	0.041	0.928	–0.022	0.962
Total SCOPA-Cog	–1.083	0.390	–0.832	0.490

*^*a*^*p-*value taken from GLM without adjustment for covariates.*

*^*b*^*p-*value taken from GLM adjusted for DBS, MDS-UPDRS III, age at time of assessment, age of onset and disease duration.*

*ACE-R, Addenbrooke’s Cognitive Examination – Revised; SCOPA-Cog, Scales for Outcomes in Parkinson’s Disease Cognition.*

## Discussion

Previous studies have indicated a link between cholesterol levels and metabolism, and age-associated cognitive impairment and dementia. Sex-specific differences in cholesterol levels complicate studies on cognitive performance, with only a few studies considering males and females separately (Walhovd et al., 2014; Bates et al., 2017; Zhao et al., 2019) or controlling for sex as a covariate (Reitz et al., 2010; Crichton et al., 2014; Sterling et al., 2016; Svensson et al., 2019; Choe et al., 2021). In the current study, females with PD showed higher LDL, HDL, and TC levels when compared to males with PD. Such differences are consistent with studies on healthy aged populations (Bates et al., 2017; Zhou et al., 2018; Zhao et al., 2019) and individuals with PD (Sterling et al., 2016). However, the current study is the first to investigate sex-specific differences in lipids and cognition in people with PD, measuring multiple lipid fractions and using two validated cognitive assessment protocols.

The present study reports a novel association between higher HDL cholesterol levels and poorer cognitive function among females with PD, but not males. Furthermore, in females, HDL cholesterol was associated with poorer performance in all global and domain-specific assessments of cognition except one, being total SCOPA-Cog, total ACE-R, and ACE-R subdomains of attention and orientation, memory, fluency and language. Such consistent associations are suggestive of a robust association between HDL cholesterol and cognition in females with PD. However, conflicting findings exist within this area of research, as multiple healthy elderly cohort studies report that plasma HDL levels are protective and are associated with retained cognitive function (Van Exel et al., 2002; Reitz et al., 2010; Crichton et al., 2014; Bates et al., 2017; Kinno et al., 2019; Svensson et al., 2019), while others exhibit no link between HDL levels and incidence of cognitive impairment and dementia (Yaffe et al., 2002; Li et al., 2005; Zhou et al., 2018). Our findings therefore point to a disease-specific influence of serum cholesterols on cognition. Coupled with the current finding of a sex-specific influence of HDL on cognition in a cohort of PD, serum cholesterols appear to be mediators of cognition in PD, though the disease-specific and sex-specific mechanisms are still unclear.

As aforementioned, sex is an important factor to take into consideration when studying cognitive ability (Song et al., 2014; Nicoletti et al., 2017; Cholerton et al., 2018; Reekes et al., 2020; Bakeberg et al., 2021). In general, it is widely accepted that non-verbal and verbal reasoning skills such as language and fluency, decision-making and memory are cognitive strengths of females when compared to males in healthy cohorts (Li and Singh, 2014), and among people with PD (Bakeberg et al., 2021), which may be the result of sex-specific structural and functional connectivity networks (Lin et al., 2018). Such sex-specific differences in neurobiology likely underpin the novel finding reported herein, though sex-specific mechanisms underlying the differential influence of lipid profile on cognition are still largely unknown. Literature suggests a number of potential mechanisms, including sex differences in lipid transportation and age-related lipid changes (Knopp et al., 2006; Casiglia et al., 2008), as well as genetic differences in lipid metabolism and steroid hormone synthesis in males and females (Sowers et al., 2006), and an association between elevated cholesterol levels and α-synuclein-related cognitive impairment (Allinquant et al., 2014; Lu et al., 2017; Jin et al., 2019). Notably, another plausible mechanism relates to the neuroprotective effects of female sex hormones, outlined below.

Within this cohort, our findings were specific to females likely to be in the peri- or post-menopausal period. While the neuroprotective properties of oestrogen are well-established (Miller and Cronin-Golomb, 2010; Lin et al., 2018), such effects are known to be altered during the peri-menopausal transition, and recent studies indicate that the transition to menopause can also trigger chronic low-grade inflammation (Yin et al., 2015; El Khoudary et al., 2018) and elevated systemic levels of inflammatory cytokines (e.g., IL-2, IL-4, and IL-6) (Giuliani et al., 2001; Yasui et al., 2007; Mishra and Brinton, 2018). Additionally, it is increasingly thought that low-grade, chronic inflammation occurs within people with PD, and that it is central in the genesis and pathophysiology of PD (Houser and Tansey, 2017; Gorecki et al., 2019, 2020). Importantly, it has been reported that the generally accepted “good” HDL cholesterol may become dysfunctional in instances of elevated inflammation, losing its anti-inflammatory and cardioprotective properties (Van Lenten et al., 2001; Ansell et al., 2007; Navab et al., 2009; Hb et al., 2011; El Khoudary et al., 2018; Nazir et al., 2020). As such, we propose that within females in this PD cohort, HDL cholesterols may have become dysfunctional and lost their protective effects. While the current study reports an inverse association between HDL-cholesterol and cognitive performance in females, Bates et al. (2017) reported a positive association between HDL-cholesterol and cognition, though this was in healthy ageing females (Bates et al., 2017). We believe that the inflammatory state, and resultant dysfunctional HDL cholesterol, proposed in individuals with PD, and to a greater degree in females with PD, may provide an explanation for these contradicting findings. Moreover, given their propensity to an enhanced peri- and post-menopausal inflammatory state, females with PD may lack the positive HDL effects to a greater degree than their male counterparts. Such literature is often overlooked and should be considered in future studies assessing markers of inflammation coupled with lipidomic profiles and cognition in PD cohorts.

Contrasting with the strong association between HDLs and cognitive performance in females with PD in the current study, associations between cognition and TC or LDL levels were less robust. While LDL levels were associated with poorer performance in the visuospatial domain in females, no significant associations were found for any other cognitive domains, or among males with PD. Conversely, one other study investigating serum cholesterol in people with PD found executive function and fine motor control were significantly associated with LDL cholesterol in people with PD, but not healthy controls (Sterling et al., 2016), whereas other studies in healthy populations do not report significant findings between cognition and LDL levels (Rej et al., 2016) or cholesterol-lowering medications (Giugliano et al., 2017; Gencer et al., 2020). Thus, the current study adds to a growing body of literature indicating that serum lipid profiles may mediate cognitive ability differently in healthy and diseased states.

## Limitations

A number of limitations of the current study must be noted. Firstly, participants were assessed in two different movement disorder centres, however, possible scoring variability was mitigated by the use of standardised clinical assessment protocols which were administered by trained clinician-researchers. Secondly, the home-based recruitment excluded patients with more advanced PD, which may contribute to higher cognitive scores among the current cohort that do not represent the full spectrum of cognitive impairment in PD. Thirdly, as the study was cross-sectional in nature, the relationship between changes in cholesterol levels and cognitive decline over time was not assessed. Furthermore, other vascular risk factors, menopausal state/hormone status, and inflammatory marker information was not available for analysis. Therefore, the present findings should be confirmed using more comprehensive cognitive testing protocols in larger longitudinal studies, to further examine how vascular and inflammatory risk factors and cholesterol levels associate with disease course in PD. Lastly, the effects of cholesterol-lowering medications were not accounted for in the current study due to a lack of retrospective availability of information regarding the use of non-PD medications among participants. However, statins are known to primarily influence LDL levels and to have only relatively minor effects on HDL levels (Chapman, 2004; Baigent et al., 2010). Moreover, recent studies, as well as a very large longitudinal systematic review, do not report a significant influence of LDL cholesterol-lowering medications on cognitive status nor risk of cognitive decline and dementia (McGuinness et al., 2016; Giugliano et al., 2017; Gencer et al., 2020). Therefore, the findings reported herein concerning HDL levels and cognitive impairment in females with PD are considered valid. However, further studies are required to confirm the present findings, and to investigate the mechanisms underlying the specific association of HDL-cholesterol levels and poorer cognitive performance in females with PD.

## Conclusion and Future Directions

While the sex-specific association between HDL and cognition evident here may be explained by peri-menopausal changes in inflammatory status and altered HDL properties, this requires further study. Furthermore, inflammation is known to induce cholesterol oxidation and production of cholesterol aldehydes (Wentworth et al., 2003; Bosco et al., 2006), such as the blood brain barrier-traversing metabolite 27-hydroxycholesterol (27-OHC) (Bosco et al., 2006; Shafaati et al., 2011; Marwarha and Ghribi, 2015; Schommer et al., 2018), which may be relevant in this context, as 27-OHC has been shown to increase α-synuclein levels, as well as aggregation and fibrilization of the protein (Bosco et al., 2006; Koob et al., 2010; Thanan et al., 2014; Marwarha and Ghribi, 2015; Schommer et al., 2018). As the accumulation of α-synuclein in the cortex is the hallmark of PD and has long been associated with cognitive decline in people with the disease (Braak et al., 2003; Lewis et al., 2010; Diógenes et al., 2012; Twohig and Nielsen, 2019; Cinar et al., 2020), future studies should examine circulating levels of this metabolite and α-synuclein levels in relation to cognition to elucidate whether this underpins the current findings.

The novel findings of this cross-sectional study provide further support for the role of cholesterol as a disease-modifying factor for cognitive dysfunction within PD (Ferri et al., 2005; Cheng et al., 2014; Cooper et al., 2015), supporting the notion that elevated levels of cholesterol may aggravate the pathophysiology of the disease (Paul et al., 2015, 2017; Jin et al., 2019). Furthermore, the identified sex-specific effect of HDL in relation to cognition suggests the presence of differing mechanisms underlying cognitive decline in males and females with PD. Vascular factors, including lipid profile, may be a relevant disease-modifying factor for cognition in females with PD, and therefore present as modifiable lifestyle factors. Future studies should therefore consider dietary interventions to delay, slow or prevent the progression to cognitive impairment and dementia in females with PD. In light of the robust association between HDL levels and cognition in females with PD, further studies should investigate the underlying mechanisms in order to maximise the therapeutic and diagnostic potential of these findings.

## Data Availability Statement

The raw data supporting the conclusions of this article will be made available by the authors, without undue reservation.

## Ethics Statement

The studies involving human participants were reviewed and approved by Sir Charles Gairdner Hospital Human Research and Ethics Committee (Approval number 2006/073). The patients/participants provided their written informed consent to participate in this study.

## Author Contributions

MCB executed the research project, designed and executed the statistical analysis, and wrote the first draft of the manuscript. AG executed the research project, and reviewed and critiqued the statistical analysis and manuscript preparation. JK executed the research project, and reviewed and critiqued the manuscript preparation. AJ and SM organized and executed the research project. MB executed the research project. SG and MH conceptualised the research project and reviewed and critiqued the manuscript preparation. RS reviewed and critiqued the manuscript preparation. SW organized the research project. FM conceptualised the research project, and reviewed and critiqued the statistical analysis and manuscript preparation. RA conceptualised and organized the research project, designed the statistical analysis, executed the statistical analysis, and reviewed and critiqued the manuscript preparation. All authors contributed to the article and approved the submitted version.

## Conflict of Interest

The authors declare that the research was conducted in the absence of any commercial or financial relationships that could be construed as a potential conflict of interest.
